# Ambiguous genes due to aligners and their impact on RNA-seq data analysis

**DOI:** 10.1038/s41598-023-41085-6

**Published:** 2023-12-08

**Authors:** Alicja Szabelska-Beresewicz, Joanna Zyprych-Walczak, Idzi Siatkowski, Michał Okoniewski

**Affiliations:** 1 Department of Mathematical and Statistical Methods, Poznan University of Life Sciences, Wojska Polskiego 28, 60-637 Poznan, Poland; 2 Scientific IT Services, ETH Zurich, Weinbergstrasse 11, 8092 Zurich, Switzerland

**Keywords:** Next-generation sequencing, RNA sequencing, Applied mathematics, Computational science, Scientific data, Statistics

## Abstract

The main scope of the study is ambiguous genes, i.e. genes whose expression is difficult to estimate from the data produced by next-generation sequencing technologies. We focused on the RNA sequencing (RNA-Seq) type of experiment performed on the Illumina platform. It is crucial to identify such genes and understand the cause of their difficulty, as these genes may be involved in some diseases. By giving misleading results, they could contribute to a misunderstanding of the cause of certain diseases, which could lead to inappropriate treatment. We thought that the ambiguous genes would be difficult to map because of their complex structure. So we looked at RNA-seq analysis using different mappers to find genes that would have different measurements from the aligners. We were able to identify such genes using a generalized linear model with two factors: mappers and groups introduced by the experiment. A large proportion of ambiguous genes are pseudogenes. High sequence similarity of pseudogenes to functional genes may indicate problems in alignment procedures. In addition, predictive analysis verified the performance of difficult genes in classification. The effectiveness of classifying samples into specific groups was compared, including the expression of difficult and not difficult genes as covariates. In almost all cases considered, ambiguous genes have less predictive power.

## Introduction

### Background

High-throughput sequencing (HTS) technology has undergone rapid and impressive development in recent years, changing the way life science research is conducted. Decreasing costs have brought HTS technology into the mainstream. HTS has led to an explosion of knowledge in genetics and genomics through the development of specific applications (whole genome sequencing and targeted sequencing) and is now being used in a growing number of biological applications, the so-called “-seq” experiments. To name a few: DNA-seq^1^, ChIP-seq^2^, RNA-seq^3–5^, BS-seq^6^. These techniques have already found many applications outside the research field, notably in various aspects of personalized medicine^7^. Here, there is still a growing need for better ways of diagnosis in order to design personalized health plans to help patients to be treated with precision, to reduce risks and also to take preventive measures. Especially in personalized medicine, results must be highly comparable and reproducible. There are many studies on the reproducibility of high-throughput RNA sequencing across different pipelines (different mappers, selection methods or laboratories). The GEUVADIS consortium (Genetic European Variation in Disease, a European Medical Sequencing Consortium) focuses on the standardization of next-generation sequencing technologies^8^. The consortium initiated a large-scale RNA-seq analysis in which data production was distributed among several laboratories. In this study, they concluded that large RNA-seq projects could be distributed across laboratories “if appropriate standardization and randomization procedures are in place. Another aspect of the reproducibility of high-throughput RNA sequencing data becomes apparent when data are integrated across laboratories.

It was found that commonly used normalization methods lead to artifacts that cause housekeeping genes to be inferred as differentially expressed^9^. A step-by-step guide was outlined to identify sources of unwanted variation in the data and to apply a workflow to remove this variation prior to differential expression analysis. As mentioned above, reproducibility of results by using different pipelines is very important in the case of extensive lists of differentially expressed genes. However, for site-to-site comparisons of the genes for which significant differential expression was identified, this agreement ranged from 70 to 76%, depending on the methods used^10^. The recent US FDA MAQC-III/SEQC consortium also investigated the accuracy, reproducibility and information content of gene product expression profiling by NGS (RNA-seq). It was shown that there is no gold standard for expression profiling and that all technologies (NGS, microarrays and qPCR) should be considered as complementary with their own strengths and limitations^11^. The SEQC study defined a number of quality metrics at the gene/transcript level and found that the majority of genes/transcripts do not meet all of these criteria at once. In terms of absolute expression profiles, this is partly due to the sampling nature of NGS and library preparation effects, but the exact sources of difficulty in accurately measuring the expression profiles of some genes are not known. It is therefore crucial to identify these genes and understand what is causing these difficulties. This may be particularly important if these genes are involved in disease, as misleading results may lead to misunderstanding of the cause of certain diseases and thus to inappropriate treatment.

A very important aspect of the reproducibility of results is the initial alignment process. The aspect of comparing reproducibility and the impact of the alignment process on further analysis has been the subject of a large number of publications. Recently, the challenges of aligning RNA-seq reads to a reference genome have been discussed, in particular the handling of spliced junctions—different aligners may handle these spliced junctions differently, leading to variations in results^12^. It has also been noted that RNA-seq alignment tools typically have a user-defined threshold for tolerating mismatches in the alignment, although it is also important to distinguish between sequencing errors and true variation between transcripts, as in some cases reads may contain mismatches caused either by sequencing errors or biological variation due to mutations^12,13^. It has been argued that the differences depend on the specific requirements of the research project, including considerations of accuracy, run time, memory usage, output format, and specific features such as handling multi-reads or splice junctions, which may influence the choice of aligner. Conclusions from the majority of studies provide evidence that the choice of aligner has an impact on the results of the analysis. For example, the performance of aligners was found to vary significantly, e.g. HISAT2 often misaligned reads to genomic locations corresponding to retrogenes^14^. Misalignment of these regions can lead to inaccurate gene expression estimates, which can affect downstream analyses and interpretations. The influence of RNA-seq algorithms (such as the alignment process) on gene expression estimation has also been investigated in terms of accuracy, precision and reliability of gene expression estimation, showing that these metrics can be significantly affected by the choice of aligner^15^. Different alignment methods—Cufflinks, RSEM and HTSeq—have been compared in terms of their ability to deal with multi-hit information at the quantification stage and which of them dealt with this issue better than others^16^. Three metrics have been used to assess the accuracy of gene expression estimates: the accuracy of gene detection, the number of incorrectly quantified genes, and the number of genes with incorrectly estimated fold change. It has been recommended to use specific aligners that meet certain criteria according to the measures introduced for gene expression estimation. Hints for aligners that give better results for the simulated data can also be found. In the context of short and long reads, the authors discuss the challenges of aligning RNA-seq reads to a reference genome.

Most of the aforementioned research focuses on comparing aligners in various aspects, but does not discuss the influence of differential analysis in the context presented in this paper.

### Motivation

The understanding and definition of difficult genes (DGs) is context specific. For example, one definition that can be found considers as difficult those genes whose expression may not follow the actual abundance of sequencing reads due to structural artifacts that are misleading for quantification algorithms^17^. It has been shown that such genes exist, that they are numerous, and that they are important for various types of biological processes and diseases, so that the misleading information about their expression can be an obstacle to obtaining correct biological knowledge. Many of these genes have been implicated in human disease. Some general characteristics of the problematic genes were sought: minimum, maximum and average exon length, total number of exons, transcript length or GC content, but no clear conclusion was reached. Another research shows that HISAT2 tends to misalign reads to pseudogenes^14^. Pseudogenes are sequences in the genome that resemble known genes but are not functional. They can arise from several processes: gene duplication, retrotransposition, and inactivation. Pseudogenes can cause problems in aligning genes to the genome because of their high sequence similarity to functional genes. It can be a challenge to determine whether a read is from the functional gene or the pseudogene. The authors discuss the relationship between the HISAT2 and STAR aligners and pseudogenes. In our research, we are extending this investigation to other aligners.

In the light of the above studies, and particularly motivated by the work of Roberts and Watson and Raplee et al.^14,17^, we have investigated whether RNA-seq analyses can be readily reproduced across different mappers.

## Results

### Review of DGs

**Figure 1:**
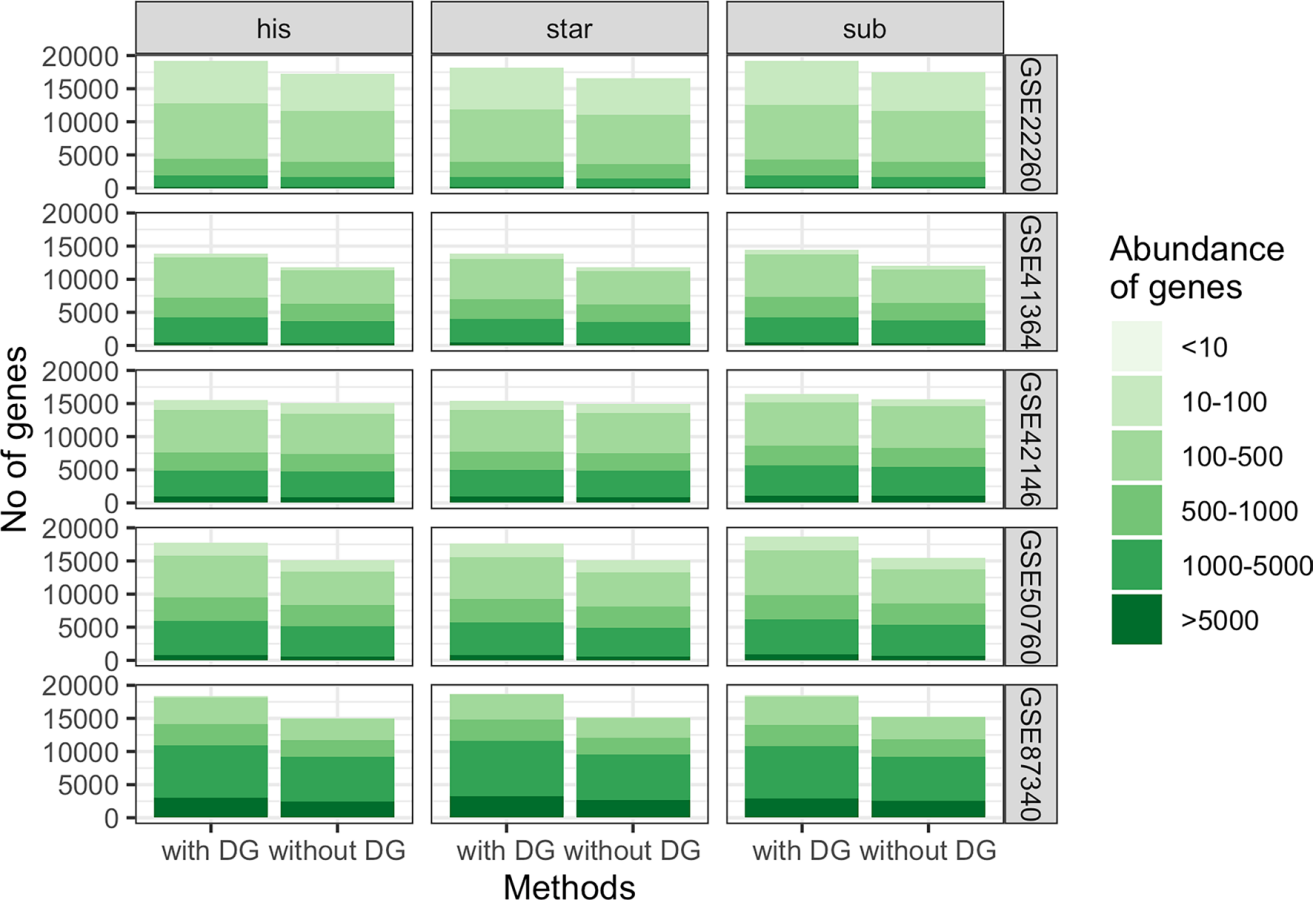
Barplot of gene abundance measured by the mean value of counts for all genes and excluding DGs for each dataset and mapper. The colours represent different levels of abundance of gene counts.

DGs were identified for each dataset using the DG search method presented in the “Material and methods”. The abundance of genes for each considered dataset and mapper with and without DGs is shown in Fig. 1.

The median library sizes for the datasets vary between 12 million reads for dataset GSE22260, 24–30 million reads for datasets GSE41364, GSE42146 and GSE50760 and over 70 million reads for dataset GSE87340 (Supplementary Figure 1S). Each dataset has a similar distribution of counts. However, the first two datasets have a lower number of highly abundant genes. The overall distribution of counts for each mapper and each dataset remains similar when DGs are excluded.

The characteristics of DGs as well as the differentially expressed genes (DEGs) that were not DGs for each dataset are presented in Table 1.

**Table 1:** Characteristics of DEGs genes found in each dataset with partitioning into DGs and DEGs that are not DGs.

Dataset	Type	Ex. min	Ex. max	Ex. median	Tr. length	Ex. no	Tr. no	Pseudogenes (%)	Coding (%)
GSE22260	DG	251	506	335	764	1	1	58	23
	Non DG	78	483	156	994	4	5	4	75
GSE41364	DG	132	492	242	780	2	1	39	29
	Non DG	80	473	156	930	4	4	8	70
GSE42146	DG	285	480	341	678	1	1	57	15
	Non DG	81	469	159	897	4	4	9	67
GSE50760	DG	140	475	252	744	2	1	42	25
	Non DG	76	478	154	1008	4	5	4	78
GSE87340	DG	169	484	274	757	2	1	55	26
	Non DG	82	487	161	960	4	4	5	68

Columns represent *Ex. min* the length of the shortest exon, *Ex. max* the length of the longest exon, *Ex. median* the median exon length, *Tr. length* the transcript length, *Ex. no* the number of exons in each transcript in each gene, *Tr. no* the number of transcripts in each gene, *Pseudogenes* the percentage of pseudogenes, and *Coding* the percentage of coding protein in each case considered. Each value was calculated as a median across all transcripts and then all genes in the datasets and considered types of difficulty.

The number of transcripts and exons for DGs is systematically smaller than for the rest of the DEGs. This translates into characteristics related to exon and transcript lengths. Namely, genes with more transcripts and exons also have greater variation in minimum, maximum and average exon lengths and greater transcript lengths within genes. The significantly higher number of pseudogenes in groups of DGs may be related to this fact. Because of the similar sequences in these types of genes to other genes, mappers have difficulty in matching pseudogenes.

#### How the counts for DGs look like?

**Figure 2:**
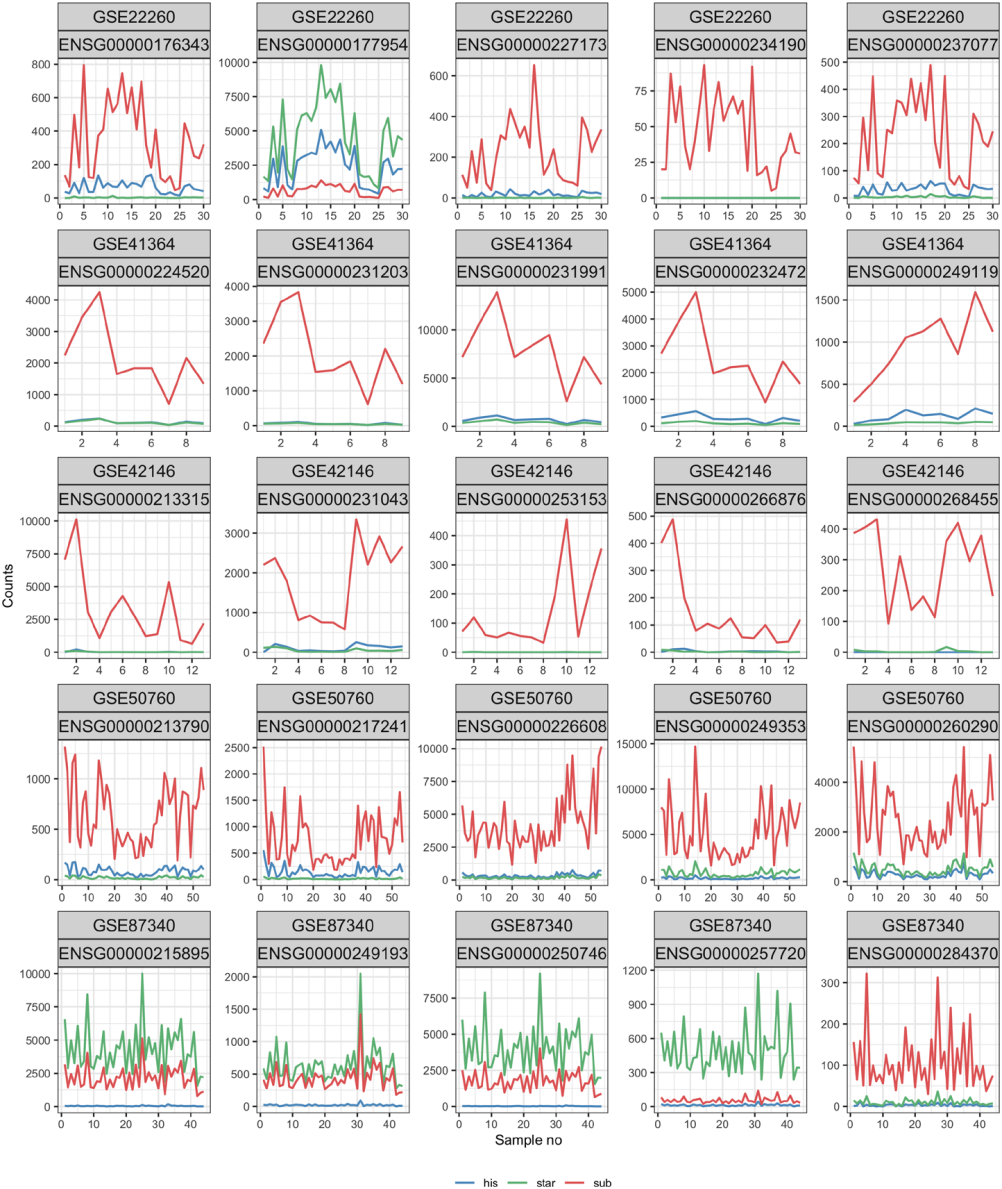
Number of read counts of exemplary DGs for each dataset. For each exemplary gene and dataset, read counts are presented for each mapper. The genes presented have the most significant statistics due to mappers and groups.

DGs are ordered first by the adjusted p values obtained for the mappers, and then by the adjusted p values for the groups. The number of counts for each sample in each dataset and for each mapper for DGs with the lowest p values are shown in Fig. 2. In most cases, the subread mapper results in significantly different counts for the genes considered. From the assumptions of our statistical model and from these plots we can conclude that the difficulties of the genes could be caused by the differences in the Subread procedure to align pseudogenes.

#### What is the size of DGs in the datasets?

To verify the impact of DGs, we next performed a differential analysis for each dataset and each mapper separately. Then we checked which DGs are present in which dataset. Table 2 summarizes the percentage of DGs compared to each dataset size as well as compared to the number of differentially expressed genes (DEGs).

**Table 2:** Percentage of significant genes due to mappers and groups across each dataset.

Datasets	Mappers					
	Hisat2		STAR		Subread	
	% of all genes	% of DEG	% of all genes	% of DEG	% of all genes	% of DEG
GSE22260	0.10	17.93	0.25	27.11	0.20	22.75
GSE41364	10.08	18.04	9.90	17.67	11.40	20.63
GSE42146	0.44	4.81	0.44	4.22	0.77	7.27
GSE50760	11.73	20.70	11.61	20.43	14.52	25.92
GSE87340	11.60	23.38	13.34	26.19	12.62	25.39

In the datasets GSE41364, GSE50760 and GSE87340, the three aligners Hisat2, STAR and Subread show relatively similar performance in identifying difficult genes, with percentages around 10 for all genes and around 20–25 for DEGs. GSE22260 has similar percentages for DEGs. However, this dataset and the GSE42146 dataset have lower percentages for all genes. In addition, the GSE42146 data set stands out with percentages for DEGs. The mechanism leading to a lower number of difficult genes or a lower number of differentially expressed genes in these particular datasets may be complex and may involve the level of gene coverage, the library sizes of the samples, as well as the experimental design used and the characteristics of the genes driven in these experiments.

#### What is the coverage profile for DGs?

**Figure 3:**
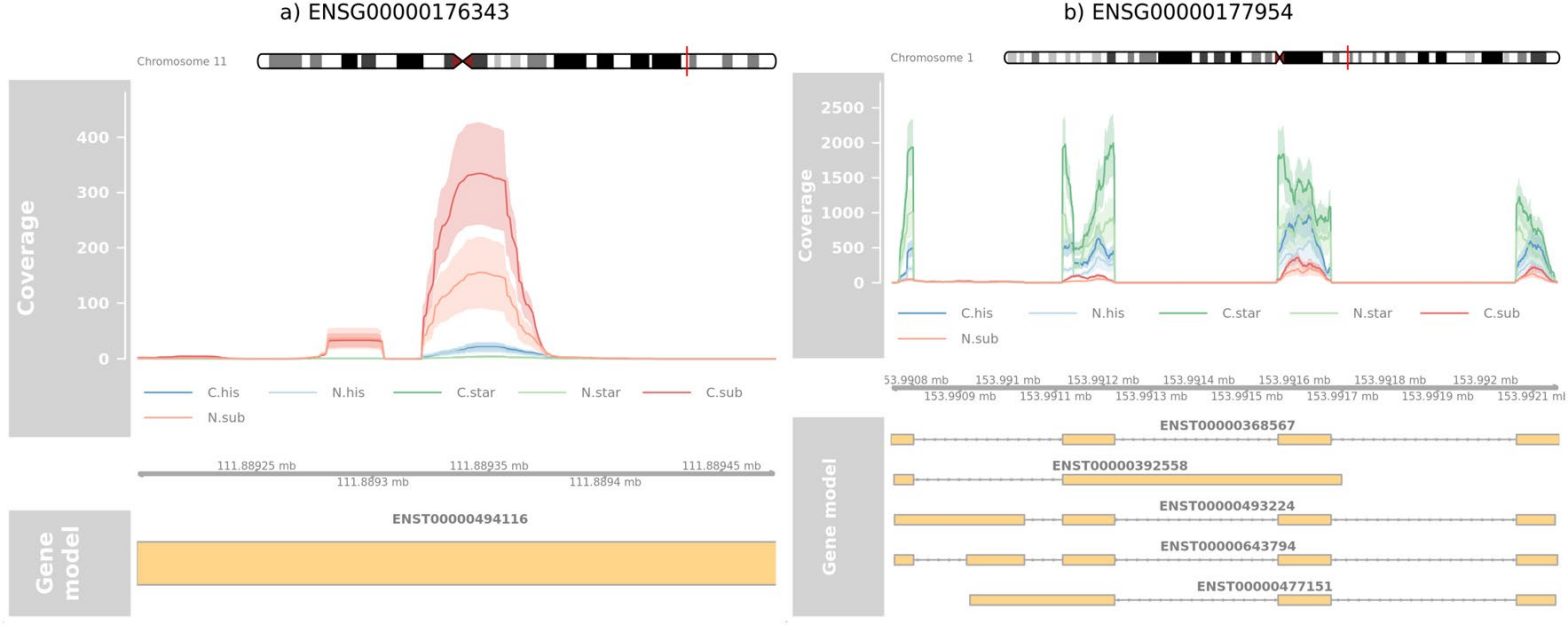
Coverage of two exemplary DGs from dataset GSE22260. Each line represents the average coverage between samples from the considered groups. Colours are linked to the mappers and shades represent 95% confidence intervals. Part (**a**) shows a pseudogene with only one transcript and one exon. Part (**b**) shows a coding protein gene with a more complex structure.

With the information on how the counts for DGs look like (see Fig. 2), we would like to have more insight into the structure of DGs. The coverage of two exemplary DGs from dataset GSE22260 shown in Fig. 2, where one is a pseudogene and the second is a protein-coding gene, is shown in Fig. 3.

The gene shown in part (a) was chosen as a representative because most DGs have one transcript and one exon (see Table 1) just like it. The expression of this gene is only in a specific region within this exon. All mappers show some expression in the same region. However, by far the largest coverage is reported by Subread. The reason for not expressing the whole exon may be the presence of similar sequences in several regions of the genome. On the other hand, for the gene presented in part b), we can observe that mostly the first transcript is expressed. The coverage within each exon is not regular. The differences in expression for the mappers can also be observed in terms of nucleotide precision. In particular, for the first exon, STAR shows the level of expression between 1000 and 2000 reads, Hisat2 shows the expression between 100 and 500 reads, while Subread reports almost no expression for this exon. The coverage profiles for the remaining top DGs from Fig. 2 are presented in Supplementary Figure 2S–6S.

### Classification

Genes discovered by differential expression analysis can serve as important biomarkers. To keep the number of biomarkers within practical limits, it’s important to confirm the predictive power of the selected genes. Therefore, the next focus was on the influence of DGs on the classification process. For this purpose, we used machine learning methods described in the subsection “Methods for classification” in the  “Material and methods” section.

#### Number of misclassified samples

**Figure 4:**
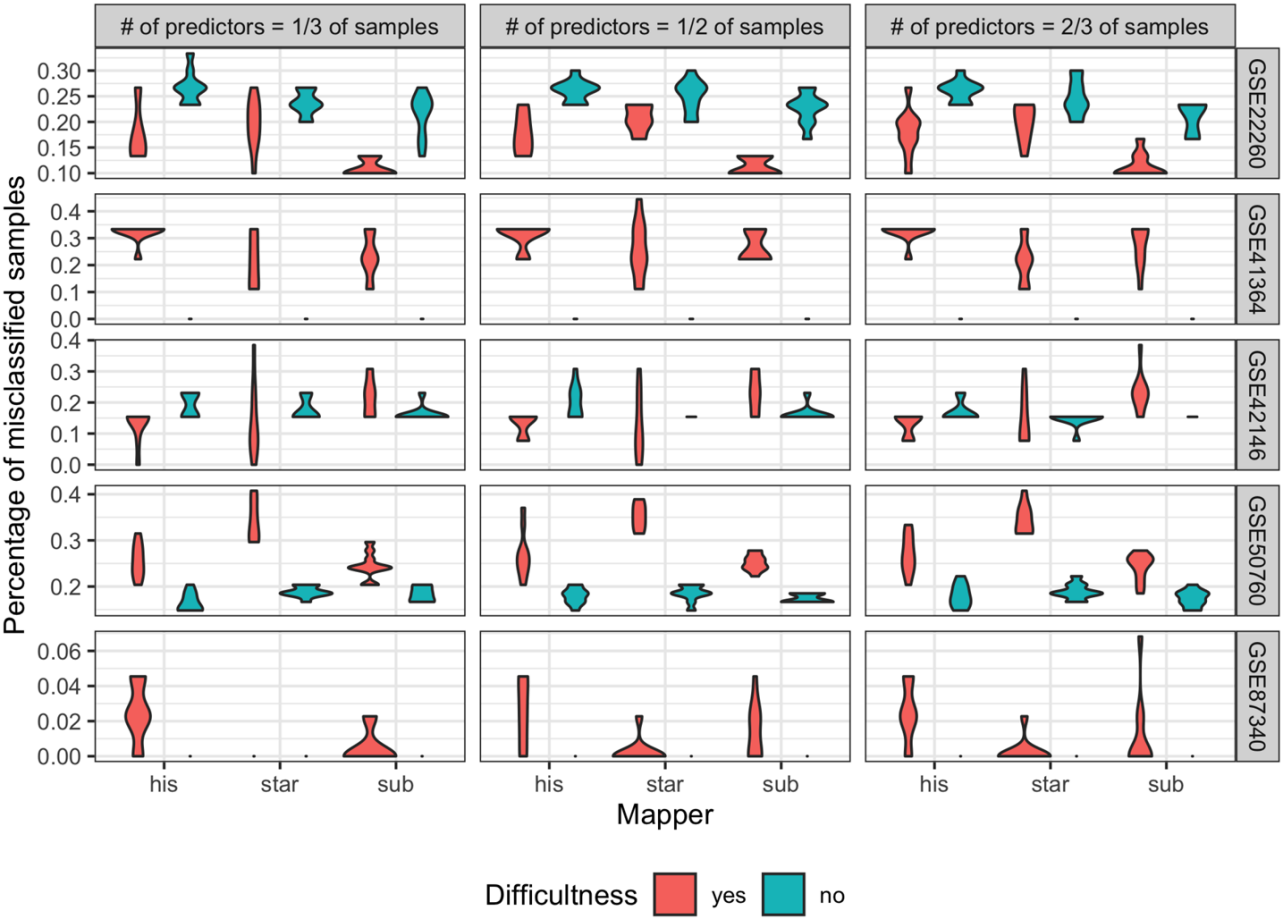
Percentage of misclassified samples for each dataset. For each dataset and number of predictors equal to 1/3, 1/2 and 2/3 of the number of samples, violin plots are drawn for 10 simulations of the joint classifier “ensemble”. The basic classifiers used in the “ensemble” classifier were: support vector machine, random forest, neural networks and rpart. The color represents difficulty cases: green color means “no” case—considering DEG that are not difficult; red color means “yes” case—considering DEG that are difficult.

For each dataset and mapper, 10 iterations of the “ensemble” classifier were performed. Difficult or not, DEGs were used as the variables used by the classifier for learning. The number of genes considered depended on the number of samples in the dataset and was 1/3, 1/2 and 2/3 of the samples, respectively. For each iteration, the number of misclassified samples was calculated using 5 or 10-fold cross-validation. The resulting misclassification percentages were presented using fiddle charts in Fig. 4.

The number of misclassified samples differs between the datasets. For dataset GSE22260 and partly for dataset GSE42146, better classification was achieved by DGs. The remaining datasets show the opposite trend. Such results can be related to the fact that these two datasets were obtained with much lower numbers of DEGs and DGs compared to other datasets.

#### AUC values

Finally, the effectiveness of the classification procedure was measured using the area under the curve (AUC) values. The mean values based on 10 iterations of the AUC were calculated for each dataset and mapper. The obtained results are summarized in Table 3.

**Table 3:** Average AUC values for considered datasets and mappers.

Dataset	No of pred	Mapper/difficultness					
		Hisat2		STAR		Subread	
		Yes	No	Yes	No	Yes	No
GSE22260	10	0.893	0.575	0.904	0.569	0.945	0.717
	15	0.901	0.598	0.884	0.636	0.939	0.668
	20	0.903	0.610	0.879	0.905	0.913	0.908
GSE41364	3	0.886	1.000	0.930	1.000	0.958	1.000
	4	0.944	1.000	0.998	1.000	1.000	1.000
	6	0.945	1.000	0.990	1.000	0.996	1.000
GSE42146	4	0.977	0.773	0.838	0.913	0.788	0.899
	6	0.988	0.795	0.970	0.908	0.874	0.894
	8	0.995	0.865	0.969	0.948	0.811	0.918
GSE50760	18	0.881	0.890	0.814	0.893	0.877	0.899
	27	0.878	0.910	0.811	0.908	0.870	0.906
	36	0.858	0.897	0.795	0.906	0.865	0.901
GSE87340	14	0.991	1.000	1.000	1.000	0.998	1.000
	22	1.000	1.000	1.000	1.000	1.000	1.000
	29	1.000	1.000	1.000	1.000	1.000	1.000

Two cases were considered: “no” case means taking into account DEGs that are not difficult; “yes” case means taking into account DEGs that are difficult. The values are mean AUC values over 10 iterations of “ensemble” classifier.

The AUC values confirm the trends in the percentage of misclassified samples. The effectiveness of the classification procedure is better for the case where we consider DEGs that are not difficult for the datasets GSE41364, GSE50760 and GSE87340. For dataset GSE22260 and partly for dataset GSE42146 a better effectiveness of the classification was obtained by DEGs that are difficult. Considering the results from Table 2 we can conclude that if the number of DGs is a relatively high proportion of all genes in the dataset then the classification procedure together with the calculation of AUC values gives better results if we exclude DGs from the analysis.

## Discussion

The aim of this paper is to present a new approach to finding DGs in the RNA sequencing experiments and to determine the properties of these genes. Roberts and Watson performed the analyses only for coding genes^17^. As recent research has suggested that some pseudogenes may have functions such as regulating gene expression^18,19^, we decided to include them in the analysis. The result is a set of DGs in which pseudogenes are significant components. In another publication, the authors have suggested that mappers may have difficulty with these types of genes (e.g. HISAT2 often misaligned reads)^14^, and our results confirm this. In our research we extended the scope with the additional Subread mapper. The results showed that this mapper tends to overestimate coverage for pseudogenes. There are a number of reasons why pseudogenes can cause problems when aligning genes to the genome. Some pseudogenes are transcribed into RNA in the same way as functional genes. This means that RNA-seq data may contain both sequences. Because of the sequence similarity between pseudogenes and their parental genes, reads can be mapped to multiple locations in the genome (multi mapping). All of these factors make it difficult to accurately quantify gene expression. Many bioinformatics tools are not designed to deal with the complexity introduced by pseudogenes. For example, some tools may discard multimapped reads, leading to an underestimation of the expression of genes with pseudogenes. These limitations highlight the importance of carefully selecting an aligner that can handle the complexity introduced by pseudogenes. In addition, the characterization of DGs in terms of number of exons and length of transcripts that were obtained is consistent with the results of the work of Roberts and Watson^17^.

By re-analyzing several public Illumina RNA-seq datasets, we have shown that these features, especially for genes that are difficult to analyze in the primary analysis, often have a significant impact on the secondary analysis and interpretation of the results. It is worth noting that the choice of aligners used was based on the universality of a considered tool and was limited only to mappers that perform alignment against the reference genome. However, depending on the biological question posed, other tools such as Salmon, Kallisto, Bowtie2 could also be used.

The magnitude of the presence of DGs can be expressed by the percentages of DGs with respect to the total number of genes and at the same time with respect to the DEG genes. In the first case, the percentages reach more than 10% for some of the datasets, while in the second case they even exceed 20% of the DEG. It is also noticeable that the Subread mapper produces very different results compared to the other two mappers. The occurrence of DGs could become a problem if the focus is on determinant analysis. If the researcher is interested in assigning samples to the correct classes (e.g. control vs. treated), he would need to use the most significant genes based on the differential analysis. As shown in our paper, such genes of interest need to be chosen carefully, as the DGs incuded may can lead to higher prediction errors.

As a practical outcome, we suggest finding difficult genes in the experiments using the methodology presented in the paper. The set of difficult genes should then be considered together with the sets of differentially expressed genes. Typical operations that can be performed are similar to those described in the paper—find the overlap of these two sets, and for example, try to perform do functional analysis and machine learning without the DG. This may allow the data analyst to avoid artifactual results and get closer to the true biological interpretation of the results.

It is crucial to identify DGs and understand the cause of their difficulties, as these genes may be involved in some diseases. By giving misleading results, they could contribute to a misunderstanding of the cause of certain diseases, which could lead to inappropriate treatment. Our research was able to point out potential DGs that are DEGs and have high differences in expression levels between different mappers. The results of the classification analysis suggest that the DGs resulted in a higher number of misclassified samples than the non-DGs. Thus, DGs have lower predictive power.

Potential directions for future work could relate to understanding the conditions under which one aligner outperforms another. This could help to develop guidelines for selecting the most appropriate aligner to use based on the specific requirements of a study. By exploring machine learning approaches, researchers may be able to automate the aligner selection process to predict the best aligner for a given dataset based on its characteristics, thereby improving our understanding of gene expression. Future research could also explore how the discrepancies in gene counts after using different aligners affect downstream analyses such as: differential gene expression, gene set enrichment analysis or network analysis. This could help researchers understand the potential impact of the choice of aligner they use. Pseudogenes are also worth investigating for further work, given their prevalence in lists of ambiguous genes. As some aligners may misalign reads to pseudogenes, future studies could focus on improving the handling of pseudogenes in RNA-seq data analysis. This could include new methods to distinguish pseudogene reads from parental genes. It is also worth investigating how many of these pseudogenes contain transposons and whether this has an effect on the misalignment of some reads for any of the mappers.

## Material and methods

### Datasets

To avoid the so-called ‘dataset bias’^20^,that some datasets are generated with specific structures and thus the results are ‘over-optimistic’ (in the case of working with our novel method), we performed the analysis in the light of several real datasets (see Table 4). We used four different datasets from the NCBI Gene Expression Omnibus (GEO) public database with accession numbers GSE22260, GSE41364, GSE42146, GSE50760 and GSE87340. The choice of datasets was mainly based on the experimental design, so that there are datasets with two and more groups, as well as with smaller and larger numbers of samples in each group. All contain human RNA-seq data for cancer-related studies.

**Table 4:** Information on selected RNA-seq datasets used in the project.

Dataset	No samples	No groups	Source
GSE22260	30	2	^21^
GSE41364	9	3	^22^
GSE42146	13	2	^23^
GSE50760	54	3	^24^
GSE87340	44	2	^25^

### Definition of ‘difficult genes’

Statistical analysis was based on a generalized linear model (GLM)^26,27^ with two factors: mappers and groups introduced into the experiment. We compared treatments, adjusting for any baseline differences between mappers and groups by fitting an additive model. As a result, we obtained the list of genes that are differentially expressed and at the same time have highly variable expression depending on the application of different alignment methods. alignment methods. The genes in these lists are considered to be DGs.

### Preprocessing pipeline

For all the datasets the same preprocessing steps were applied to minimize the sources of bias. Firstly, the quality of raw FASTQ files was checked with fastQC program (ver.0.11.5)^28^. Next, the reference three spliced aligner: Star (ver.2.5)^29^, Subread (ver.1.5.2)^30^, Hisat2 (ver.2.1.0)^31^ that perform mapping against reference genome were considered for the analysis. The choice of the aligners was dictated by the widespread use of these tools. Depending on the biological questions of interest, other aligners (e.g. those that allow alignment against a reference transcriptome) could be applied instead. Data were mapped to human genome (GRCh38/hg38) (accessed at homo_sapiens/dna/Homo_sapiens.GRCh38.dna.toplevel.fa.gz). Further, counting reads together with multi-mapping reads was done with usage of featureCounts^32^. Prior to differential analysis, each dataset was first filtered for genes with low expression. Genes with expression lower than 5 reads in more than 30% of the samples were excluded from the further analysis. To each dataset TMM normalization^33^ was applied.

### Methods for indicating DG

In this study, we analyze datasets after using different aligners. There are many existing bioinformatics approaches for RNA-Seq quantification—the conversion of raw sequencing reads into estimates of gene expression. With the mappers considered and the preprocessed data, we applied the following procedure to identify DGs.

We denote the number of reads that map to the g-th gene by $y_{gi}$. Then

1 $$\begin{aligned} {\mathbb {E}}(y_{gi}) = \mu _{gi}, \end{aligned}$$

where $i = 1, \dots , n\cdot k$, $g = 1, \dots , G$, n is the number of samples in experiment, k is the number of considered mappers and G denotes the total number of genes.

We assume that the variance can be evaluated for any value of $\mu _{gi}$. Thanks to this GLM theory can be used to fit a log-linear model:

2 $$\begin{aligned} log(\mu _{gi}) = x_{i}^T \beta _{g} + log(N_{i}), \end{aligned}$$

where $x_i$ is a vector of covariates that specifies group and mapper condition applied to sample i, and $\beta _g$ is a vector of regression coefficients by which the covariate effects are mediated for gene g. An additive model without interaction was used to find DGs. For such a model, the procedure for finding DGs is as follows:

1. Combine count tables from different mappers (Hisat2, STAR, Subread) into one table.
2. Create a design matrix using mappers and groups introduced by the experiment as factors.
3. Use the GLM implemented in the edgeR package^34^:
  - Fit a negative binomial generalized log-linear model^34^ to the read counts for each gene.
  - Run likelihood ratio tests twice: the first for the groups in the experiments and the second for the mappers.
4. Mark as DGs those that are significant (have very different expressions) due to mappers and groups.

**Figure 5:**
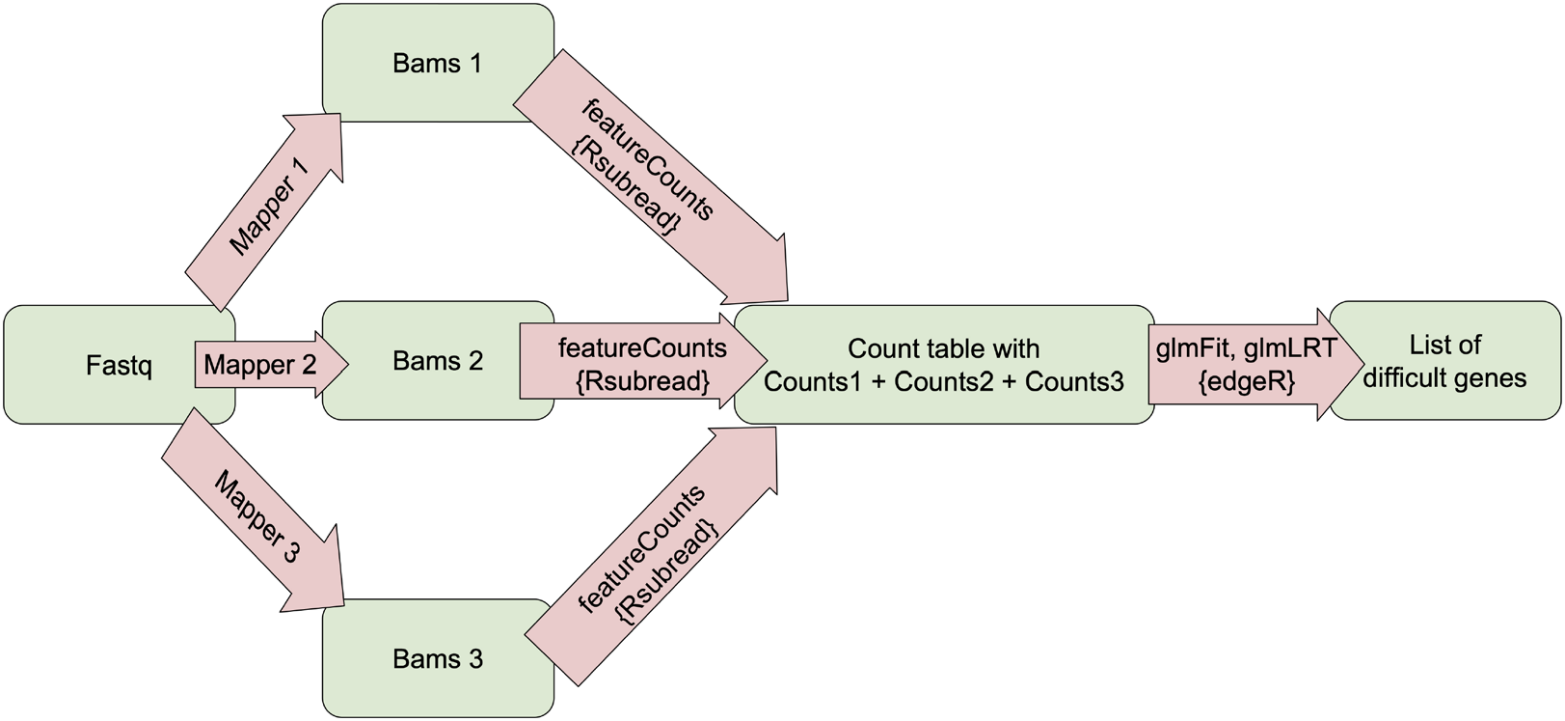
Pipeline for the procedure of seeking DGs.

First, we selected the lists of the most difficult genes, i.e. genes with the lowest $p$ value in terms of mapper and group. As a next step, we wanted to use a standard workflow that uses data for only one mapper. Therefore, for each dataset and each mapper, we determined the lists of DEGs at the $0.05$ significance level using the edgeR package. This test is based on the negative binomial distribution, which is commonly used to model the count-based data generated by RNA-seq experiments. The exact test implemented in edgeR tests for differential expression by comparing the expression levels of genes between different conditions or groups. This approach uses information from whole genes to improve the estimate of dispersion and increase the power to detect differentially expressed genes. The schematic of the overall indentification workflow is shown in Fig. 5.

As a summary of our research, we calculated percentages of the number of of significant genes with respect to the mappers and groups identified by the the experiment.

### Methods for classification

DEGs can be used as biomarkers for disease diagnosis and prognosis, facilitating personalized medicine and guiding drug development. However, for practicality and ease of testing, it’s critical to maintain a manageable number of biomarkers, which requires verification of the predictive power of selected genes. Discriminant analysis can enhance their utility by classifying observations into predefined groups based on these gene biomarkers, evaluating their performance and identifying the most influential ones for further investigation or diagnostic application. Therefore, the next step was to investigate the influence of the DGs on the classification process. For each mapper and each dataset, the top DEGs that were or were not difficult to predict whether a sample belonged to a particular group introduced in the experiment (e.g. control or treatment group) were used as features to input into the classifier. Predictions were made based on the number of top DEGs genes and top DGs corresponding to 33%, 50% and 67% of the samples in a given dataset. To reduce the bias associated with the choice of classification algorithm, the joint classifier ‘ensemble’ was used^35^. This classifier uses a number of machine learning algorithms to make predictions and then combines them to make a final prediction. In the classifier 100 bootstrap iterations were performed for four classifiers: support vector machine, random forest, neural networks and rpart. To reduce bias in the results, the classification procedure was repeated 10 times. For validation, 5-fold and 10-fold cross-validation procedures were used for datasets with less and more than 20 samples, respectively. The selected DEGs and DGs used for training and prediction in each iteration were obtained in the training step, ensuring that the model generalizes well to unseen data. The results are presented in the violin plots and the average AUC values were calculated based on the pROC package^36^.

All statistical analyses were performed using R software 3.6.2^37^. All visualisations were prepared using the ggplot2 package^38^.

### Supplementary Information

Supplementary Figures.

## Data Availability

All data used in the project are available on the NCBI Gene Expression Omnibus (GEO) public database. https://www.ncbi.nlm.nih.gov/geo/.

## Code availability

All the implementations of method steps performed in this project are available in GitHub repository https://github.com/Bioinformatics-Group-UP-Poznan/ambiguous-genes.
