# Supplementary material for: Ambiguous genes due to aligners and their impact on RNA-seq data analysis
Source: Sci Rep. 2023 Dec 8;13:21770. doi: 10.1038/s41598-023-41085-6 (PMC10709571; doi:10.1038/s41598-023-41085-6)
Supplement: Supplementary file 1 — Supplementary Figures. [file 41598_2023_41085_MOESM1_ESM.pdf]

Suplementary Material

Ambiguous genes due to aligners and their impact on RNA-seq data analysis.

Alicja Szabelska-Beresewicz[1,*], Joanna Zyprych-Walczak[1,*], Idzi Siatkowski[1], and Michał Okoniewski[2]

[1]Department of Mathematical and Statistical Methods, Poznan University of Life Sciences, Wojska Polskiego 28, Poznań, 60-637, Poland
[2]Scientific IT Services, ETH Zurich, Weinbergstrasse 11, Zürich, 8092, Switzerland
[*]aszab@up.poznan.pl; joanna.zyprych-walczak@up.poznan.pl

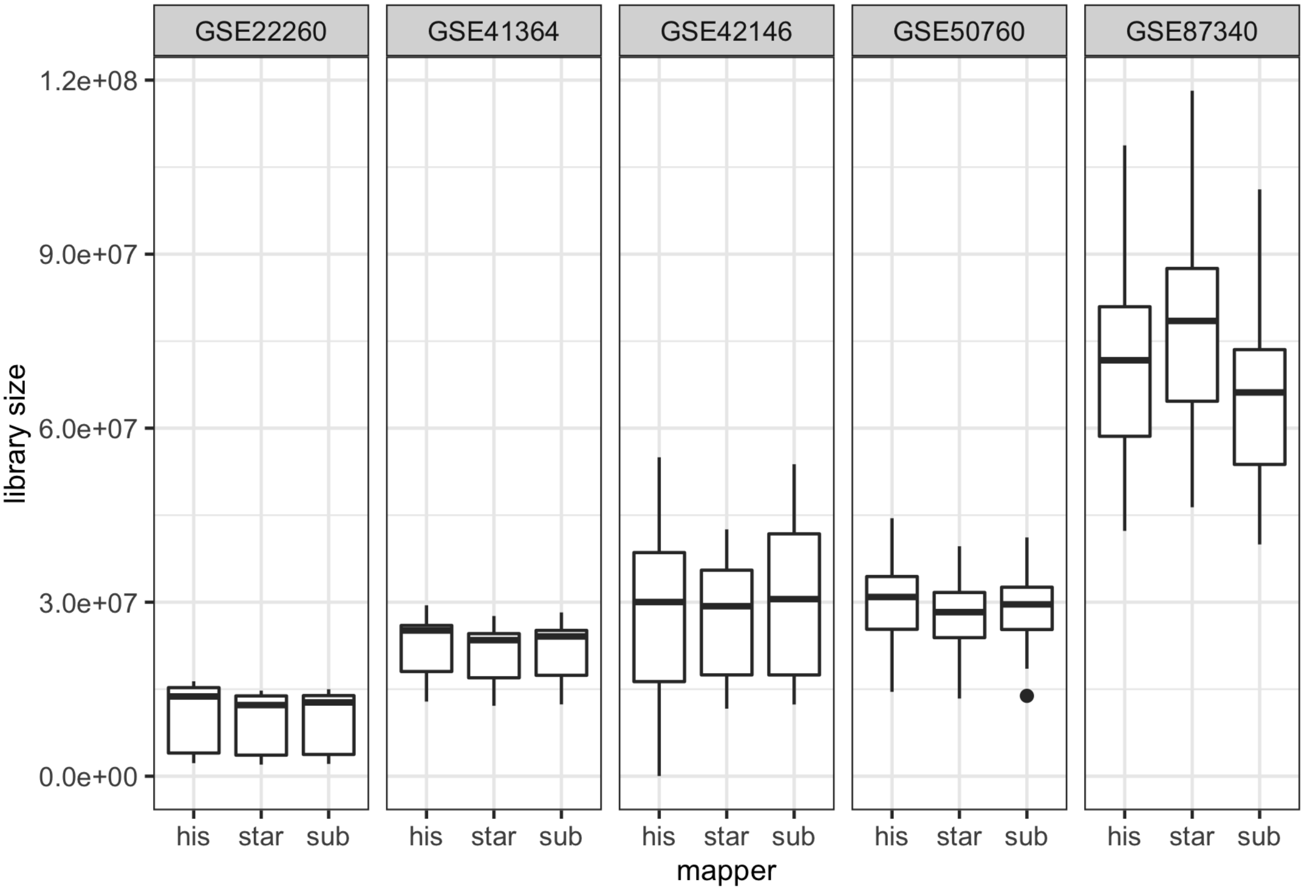

Figure 1S. Distribution of library sizes for the samples in each dataset and for each mapper.

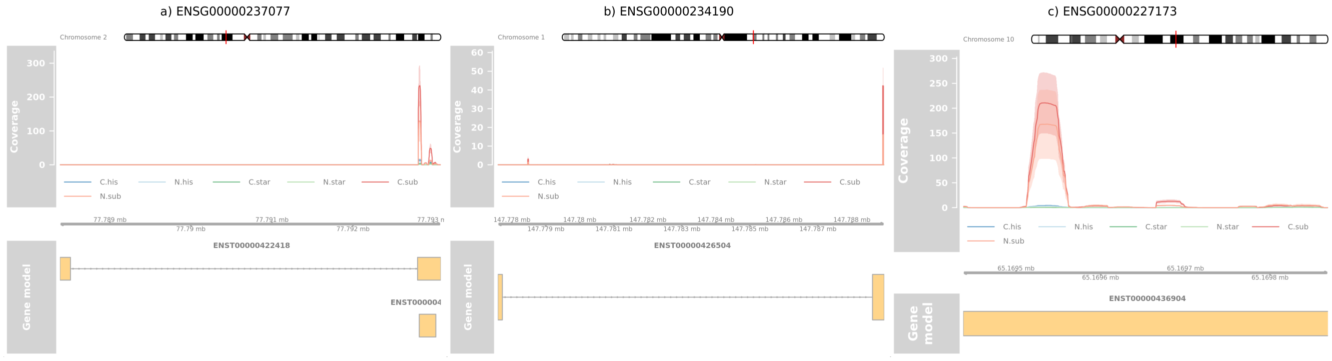

Figure 2S. Coverage of exemplary DGs from dataset GSE22260. Each line represents the average values of coverage between samples from the considered groups. The colors are linked to the mappers and shades represents 95% confidence intervals.

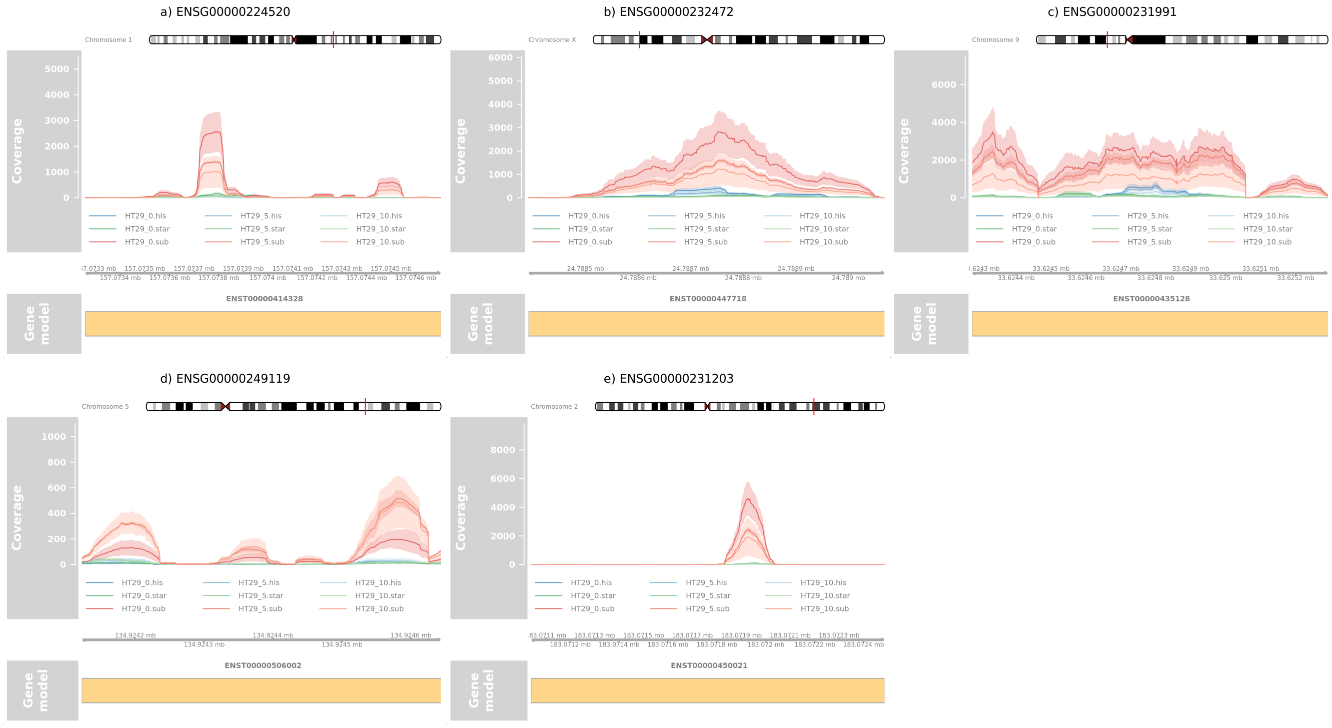

Figure 3S. Coverage of exemplary DGs from dataset GSE41364. Each line represents the average values of coverage between samples from the considered groups. The colors are linked to the mappers and shades represents 95% confidence intervals.

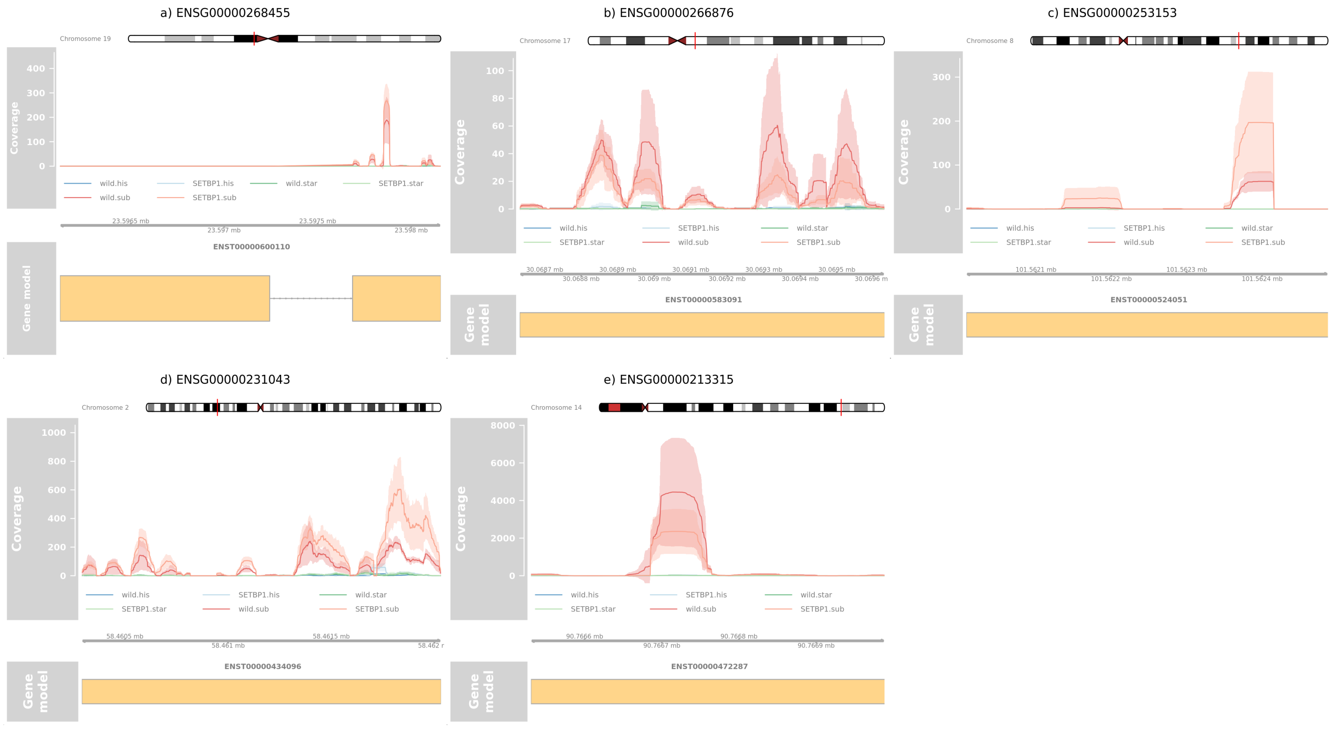

Figure 4S. Coverage of exemplary DGs from dataset GSE42146. Each line represents the average values of coverage between samples from the considered groups. The colors are linked to the mappers and shades represents 95% confidence intervals.

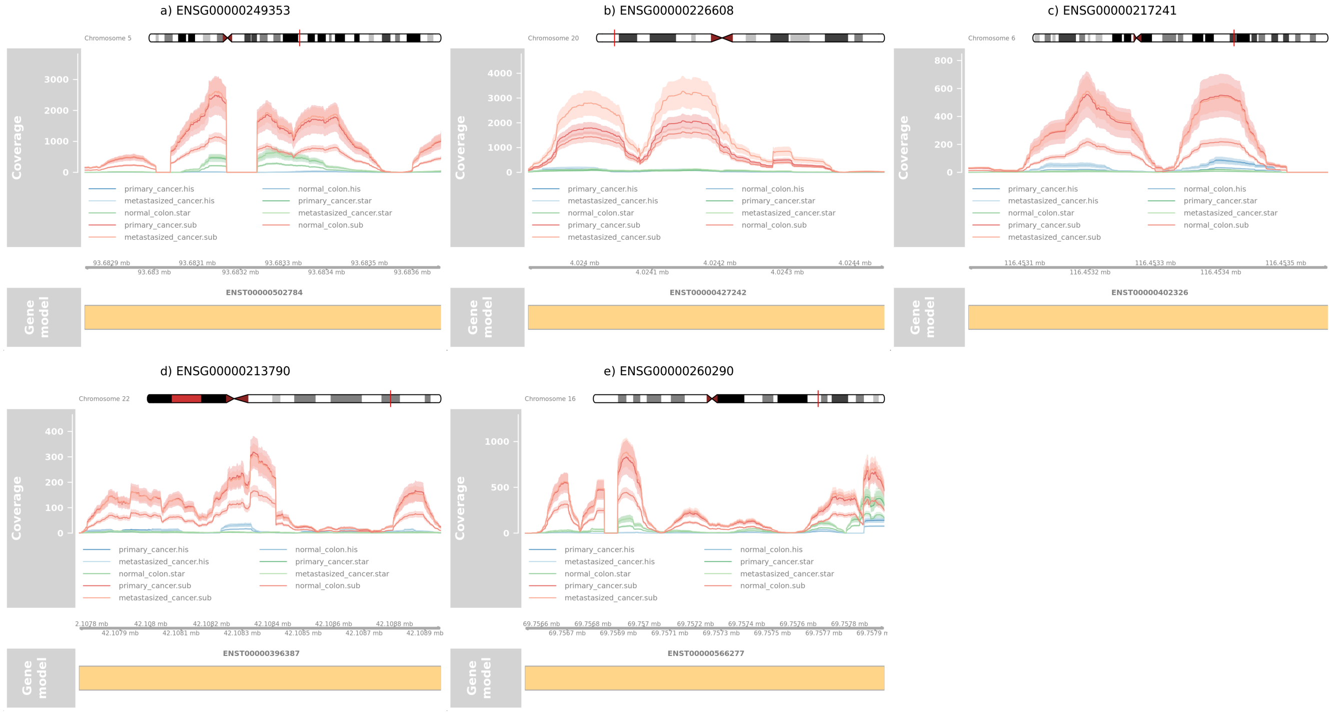

Figure 5S. Coverage of exemplary DGs from dataset GSE50760. Each line represents the average values of coverage between samples from the considered groups. The colors are linked to the mappers and shades represents 95% confidence intervals.

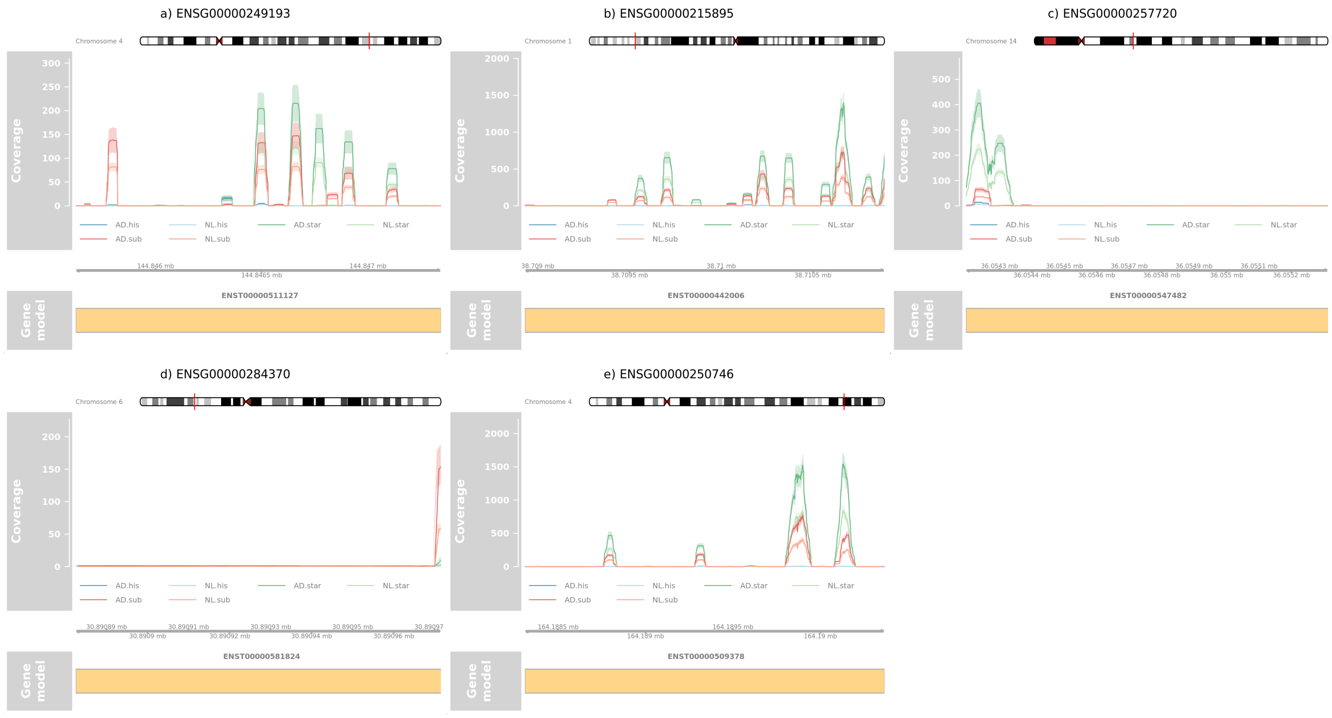

Figure 6S. Coverage of exemplary DGs from dataset GSE87340. Each line represents the average values of coverage between samples from the considered groups. The colors are linked to the mappers and shades represents 95% confidence intervals.
